# Calcium signaling in neurodegeneration

**DOI:** 10.1186/1750-1326-4-20

**Published:** 2009-05-06

**Authors:** Philippe Marambaud, Ute Dreses-Werringloer, Valérie Vingtdeux

**Affiliations:** 1 Litwin-Zucker Research Center for the Study of Alzheimer's Disease, The Feinstein Institute for Medical Research, North Shore-LIJ, Manhasset, New York 11030, USA; 2 Department of Pathology, Albert Einstein College of Medicine, Bronx, New York 10461, USA

## Abstract

Calcium is a key signaling ion involved in many different intracellular and extracellular processes ranging from synaptic activity to cell-cell communication and adhesion. The exact definition at the molecular level of the versatility of this ion has made overwhelming progress in the past several years and has been extensively reviewed. In the brain, calcium is fundamental in the control of synaptic activity and memory formation, a process that leads to the activation of specific calcium-dependent signal transduction pathways and implicates key protein effectors, such as CaMKs, MAPK/ERKs, and CREB. Properly controlled homeostasis of calcium signaling not only supports normal brain physiology but also maintains neuronal integrity and long-term cell survival. Emerging knowledge indicates that calcium homeostasis is not only critical for cell physiology and health, but also, when deregulated, can lead to neurodegeneration via complex and diverse mechanisms involved in selective neuronal impairments and death. The identification of several modulators of calcium homeostasis, such as presenilins and CALHM1, as potential factors involved in the pathogenesis of Alzheimer's disease, provides strong support for a role of calcium in neurodegeneration. These observations represent an important step towards understanding the molecular mechanisms of calcium signaling disturbances observed in different brain diseases such as Alzheimer's, Parkinson's, and Huntington's diseases.

## Calcium signaling and neuronal functions in the healthy brain

**Figure 1:**
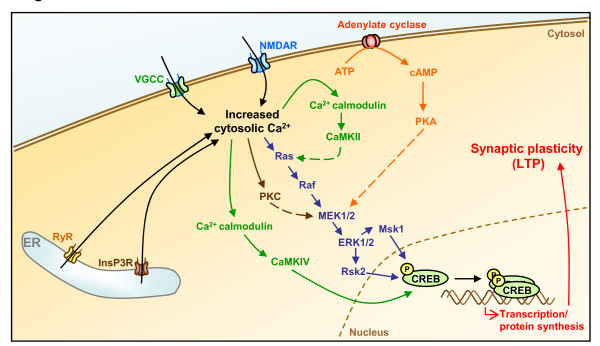
**Calcium signaling in synaptic plasticity**. Synaptic activity results in the elevation of cytosolic calcium levels by promoting extracellular calcium influx (through opening of specific cell surface calcium channels, e.g. VGCCs or NMDAR) or ER calcium efflux (via activation of RyRs or InsP3Rs). Increased cytosolic calcium concentrations initiate the activation of several kinase-dependent signaling cascades leading to CREB activation and phosphorylation at Ser133, a process critical for protein synthesis-dependent synaptic plasticity and LTP.

Brain functions are manifested at specific synapses through release of neurotransmitters inducing a number of biochemical signaling events in postsynaptic neurons. One of the most prominent of these events is a rapid and transient rise in calcium levels. This local increase in calcium concentrations results in a number of short-term and long-term synapse-specific alterations. These include the insertion or removal of specific calcium channel subunits at or from the membrane and the post-translational modification or degradation of synaptic proteins [1-3]. Beside these local events at the synapse, calcium elevation in postsynaptic neurons activates a cascade of signaling events that result in gene expression and that are essential for dendritic development, neuronal survival, and synaptic plasticity [4,5] (Figure 1).

Under resting conditions, free cytosolic calcium levels in neurons are maintained around 200 nM. Upon electrical or receptor-mediated stimulation, calcium levels rise to low micromolar concentrations by a mechanism of extracellular calcium influx or calcium release from intracellular stores. Extracellular calcium concentrations are several magnitudes higher compared to cytosolic calcium levels. Thus, calcium can enter the cells during opening of specific ion channels, which include the voltage-gated calcium channels (VGCCs) and several ligand-gated ion channels, such as glutamate and acetylcholine receptors [6,7]. The main intracellular calcium store is the endoplasmic reticulum (ER) from where calcium can be released into the cytosol via activation of the inositol 1,4,5-triphosphate receptors (InsP_3_Rs) or ryanodine receptors (RyRs) [6]. Basal cytosolic calcium levels are in part maintained by powerful calcium-binding and calcium-buffering proteins (e.g. calbindin or parvalbumin) or by active uptake into internal stores by the Sarco/ER calcium-ATPase (SERCA) at the ER membrane or by the mitochondrial uniporter [6].

### Calcium signaling and synaptic activity

Synaptic plasticity is thought to be crucial for information processing in the brain and to underlie learning and memory. Widely studied models for synaptic plasticity are long-term potentiation (LTP) and long-term depression (LTD). LTP is a cellular model underlying learning and memory, which has been described in all excitatory pathways in the hippocampus and in different other brain regions [8,9].

LTP is usually divided into three temporal phases. The first stage is initial LTP or referred as short-term potentiation (STP) and is characterized as being protein-kinase and protein-synthesis independent. The next phase is early LTP (E-LTP) and its expression is mediated by activation of various protein kinases and the insertion of glutamate receptors into the postsynaptic membrane [10,11]. The third phase is late LTP (L-LTP) and lasts from a few hours to several days and is correlated to long-term memory. The critical biochemical feature for L-LTP is a requirement for new gene expression and protein synthesis [12-14]. An essential event necessary for the induction of all types of LTP appears to be the influx of calcium into the postsynaptic spine. Indeed, LTP induction can occur when postsynaptic hippocampal neurons are loaded with calcium [15]. Conversely, LTP can be blocked with calcium chelators preventing the postsynaptic rise in calcium [15-19]. Extracellular calcium influx is not, however, the only event controlling LTP. Depletion of ER calcium stores can block LTP, suggesting that calcium release from intracellular stores is also critical for LTP induction (see next paragraph and Ref. [9]).

In the majority of synapses that support LTP, the postsynaptic increase in calcium is mediated by the *N*-methyl-D-aspartate receptor (NMDAR) [20-22]. The requirement for NMDAR activity in LTP was not only demonstrated in the hippocampus, but also in other brain regions, such as the amygdala [23] and frontal cortex [24]. In addition animal studies confirmed the importance of NMDAR for learning and memory, by showing that NMDAR inhibition blocked spatial and associative learning [25-28]. LTP induction, however, can also occur in the CA1 region as a result of L-type VGCC activation [29,30]. Furthermore, several studies have shown that intracellular calcium stores may also play a role in the increase of postsynaptic calcium levels upon synaptic activity. Indeed, depletion of ER calcium stores with thapsigargin has been shown to inhibit the induction of LTP in brain hippocampal slices [31-33] and inhibition of the ER calcium channels RyRs or InsP3Rs appeared to affect specific LTP forms [30,33,34].

### Calcium signaling and CaMKs

The rise in intracellular calcium levels upon synaptic activity triggers the activation of several kinases critical for the induction and expression of LTP. These include the calcium/calmodulin-regulated protein kinases CaMKII and CaMKIV [35], the cAMP-dependent protein kinase A (PKA) [36], PKC [37,38] and MAPK/ERKs. A broad range of evidence from molecular, cellular, and transgenic animal studies established CaMKII as a key factor in LTP. Postsynaptic injection of CaMKII inhibitors or genetic deletion of a critical CaMKII subunit blocked the ability to generate LTP and impaired learning in mice [10,37,39,40]. In addition, enhancing postsynaptic CaMKII activity in CA1 neurons was able to induce synaptic potentiation and subsequent induction of LTP [41,42]. Some of the consequences of CaMKII activation are an increase of the calcium conductance of the glutamate receptor AMPAR (α-amino-3-hydroxy-5-methyl-4-isoxazole propionic acid receptor), and this by a mechanism of phosphorylation of the channel by CaMKII and by increasing AMPAR recycling. Both of these effects represent a way to regulate the strength of glutamergic synapses [43-45].

### Calcium signaling and MAPK/ERKs

One of the protein kinase families critical for the expression of LTP is the MAP kinase family, specifically the extracellular signal-regulated kinases (ERKs). ERKs belong to the large family of mitogen-activated protein kinases (MAPKs) which have a vital role in differentiation and proliferation in dividing cells. ERKs are also highly expressed in the adult brain, specifically in mature neurons, which are terminally differentiated and do not divide [46].

The MAPK/ERK cascade involves a consecutive and sequential activation of 3 kinases. The upstream MAPK kinase-kinases Raf phosphorylate MAPK/ERK-kinases (MEKs). MEKs are dual specific kinases that trigger the activation of MAPKs by phosphorylating a threonine and tyrosine. Phosphorylation of both residues is required for ERK activation. ERK is phosphorylated during NMDAR activation in the hippocampal CA1 region during LTP [47,48] and in cultured embryonic hippocampal neurons [49], demonstrating ERK involvement in synaptic plasticity [50-53].

It appears that activation of the MAPK/ERK cascade by calcium can be mediated by different pathways. One of these pathways is Ras-dependent. Ras represents one superfamily of small GTPases, which function as a molecular switch by cycling between an inactive GDP-bound state and an active GTP-bound state. This process is facilitated by guanine nucleotide exchange factors (GEFs) for activation and GTPase-activating proteins (GAPs) for inhibition of Ras. RasGRF1, a calcium/CaM-dependent GEF, interacts with the NR2B subunit of NMDAR and mediates activation of the calcium channel and of the Ras/Raf/ERK cascade in hippocampal neurons [54,55]. A novel RasGAP, p135 SynGAP, which is highly abundant at glutaminergic synapses, is reversibly inhibited by CaMKII [56]. This inhibition could stop the inactivation of GTP-bound Ras and thus initiate MAPK/ERK cascade activation.

In addition to Ras, PKA and PKC appear to be required for MAPK activation in cell culture models and hippocampal slices [57-59]. Further experiments also showed that PKA is required for nuclear translocation of ERK. The abundant crosstalk between these different kinase pathways suggests that the ERK cascade may represent a point of convergence from several kinases activated as a consequence of LTP induction and calcium elevation in the post-synaptic terminal [9,60] (Figure 1).

Even though the ERK family comprises 5 different isoforms, ERK1 through ERK5, ERK1 and ERK2 are the most studied in synaptic plasticity. The two kinases show nearly 85% sequence identity and are both highly expressed throughout the brain and in postmitotic neurons [46,61]. In some cases, however, ERK1 and ERK2 activation is differentially regulated. ERK2, but not ERK1, is activated in the CA1 region of the hippocampus after LTP induction [47]. In rats, ERK2 was selectively activated after contextual fear conditioning [62]. Whereas genetic ablation of ERK1 has no severe general phenotypic consequences [63,64], an ERK1 knockout mouse model was found to display significant enhancement of striatum-dependent long-term memory/LTP [65]. In contrast to ERK1, ERK2 knockout animals are embryonic lethal [51]. Conditional ERK2 inactivation revealed that ERK2 deficiency in the brain results in a reduction of cortical brain thickness and in the generation of fewer neurons [66]. Together, these data clearly suggest a different role for both ERK isoforms, with ERK1 possibly playing an accessory function to ERK2 during LTP and neuronal calcium signaling.

### Calcium signaling and CREB

It is well established that changes in intracellular calcium levels in neurons are transduced into protein synthesis through activation of specific signaling pathways and transcription factors. Protein synthesis is indeed required for persistent forms of synaptic plasticity, including LTP, via potential interactions between the mTOR (mammalian target of rapamycin) and ERK pathways in hippocampal neurons [67]. One of the transcription factors involved, cAMP-responsive element binding (CREB), has been identified as critically important for protein synthesis during LTP and long-term memory formation.

The first indications for a role of CREB in synaptic plasticity came from studies with the snail *Aplysia*, which exhibits a memory-like behavior for gill withdrawal reflex known as long-term facilitation (LTF, reviewed in [68]). A couple of studies demonstrated the requirement and sufficiency of the CREB pathway for LTF [69-73]. Similar results were obtained from studies in *Drosophila *[74,75]. In rodents, CREB inactivation leads to a decrease in long-term memory [76-78].

Phosphorylation of Ser133 in CREB was identified as the key event that must occur in order for CREB to function as a stimulus-dependent transcriptional activator. After phosphorylation at Ser133, CREB recruits CREB binding protein (CBP) to act as a transcriptional coactivator [79,80]. A number of calcium-dependent signaling pathways were shown to result in the nuclear phosphorylation of CREB at Ser133. Among these are CaMKIV [81-83] and MAPK/ERKs [57,84-87]. In contrast to CaMKs, ERKs cannot directly phosphorylate CREB. Two families of related kinases were reported to translate the signal from activated ERKs to CREB. These are the ribosomal S6 kinases (RSKs) and mitogen- and stress-activated protein kinases (MSKs) [88,89]. MAPK/ERKs are coupled to CREB activation by RSK2 [84,90,91]. A role for MSK1 was also reported *in vivo *in mice where activation of MAPK, MSK1, and CREB was found to be absolutely dependent on calcium-stimulated adenylyl cyclase and PKA activities [92,93]. It appears that CaMKIV- and ERK-induced CREB stimulation follows different kinetics, with CaMKIV dominating the rapid activation of CREB, whereas MAPK/ERKs being slower, suggesting that ERKs are required for the maintenance of CREB phosphorylation and activation [94,95]. In addition, CREB activation through MAPK/ERKs seems to be connected to PKA and PKC signaling. Activation of both PKA and PKC targets ERKs and thereby phosphorylates CREB. This PKA- and PKC-dependent phosphorylation of CREB is significantly inhibited by MEK inhibition indicating that MAPK/ERKs are mediators of both signals [57].

Thus, calcium is critically involved in synaptic activity and memory formation by regulating specific signal transduction pathways that implicate key protein effectors, such as CaMKs, MAPK/ERKs, and CREB (Figure 1). Properly controlled homeostasis of this calcium signaling not only supports normal brain physiology but also maintains normal neuronal integrity and long-term cell survival.

## Calcium homeostasis perturbations in neurodegenerative diseases

**Figure 2:**
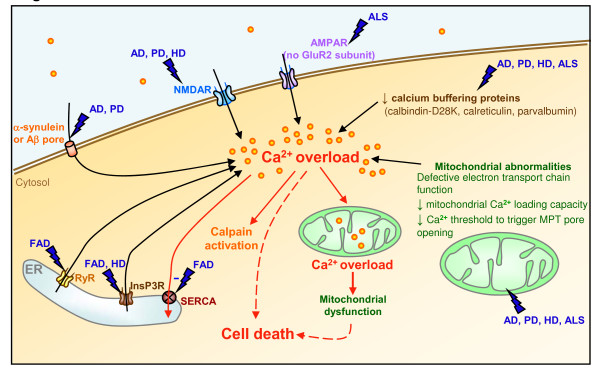
**Calcium homeostasis deregulations in neurodegenerative diseases**. AD, PD, HD, and ALS affect cytosolic calcium levels by deregulating different homeostatic control mechanisms. NMDAR or AMPAR activities, calcium buffering proteins, and mitochondrial functions were found to be deregulated in the 4 neurodegenerative conditions. α-Synuclein and Aβ peptides, the building blocks of Lewy bodies in PD and of senile plaques in AD, respectively, can form calcium-permeable ion channels at the plasma membrane. Abnormal ER calcium efflux by a mechanism of oversensitization of InsP3R (in HD and FAD) and RyR (in FAD), or of inactivation of the ER pump SERCA (in FAD), was also observed.

Perturbations in calcium homeostasis were observed in several neurodegenerative disorders including Alzheimer's disease (AD) [96-100], Parkinson's disease (PD) [101-103], Huntington's disease (HD) [104-107], and amyotrophic lateral sclerosis (ALS) [108-111] (see Table 1). Calcium homeostasis disruption implicates several mechanisms, such as alterations of calcium buffering capacities, deregulation of calcium channel activities, or excitotoxicity. Rare examples support a direct causative role of calcium homeostasis deregulation in neurodegeneration. However, compelling evidence supported by an increasing number of publications on this topic, highlights the importance of calcium deregulation in the neurodegenerative process [98,112]. We will focus in this section on how calcium homeostasis is affected in neurodegenerative disorders by taking non exhaustive examples in AD, PD, HD, and ALS (Figure 2).

**Table 1:** General description of the neurodegenerative diseases discussed in this article

**Disease**	**Clinical manifestations**	**Neuronal populations affected**	**Protein aggregates**	**Genetic factors**	**References**
Alzheimer's disease (AD)	Memory loss and behavioral abnormalities.	Loss of neurons and synapses in the cerebral cortex and certain subcortical regions.	Amyloid plaques and neurofibrillary tangles.	APP, PS1, PS2, APOE4, CALHM1...	[96-100]

Parkinson's disease (PD)	Resting tremor, postural instability, gait disturbance, bradykinesia, and rigidity.	Dopaminergic neurons of the substantia nigra pars compacta (SNpc).	Aggregated protein deposits named Lewy bodies (LB) composed of α-synuclein.	Parkin, DJ-1, PINK1, LRRK2	[101-103]

Huntington's disease (HD)	Chorea, psychiatric disturbances, and cognitive impairments.	Loss of medium spiny neurons (MSN). The area affected are mainly the striatum (caudate nucleus, putamen, and globus pallidus), and also the frontal and temporal cortex.	Accumulation and aggregation of mutant Huntingtin (containing 36 or more polyglutamine (polyQ) expansions in the N-terminal of Huntingtin).	Htt	[104-107]

Amyotrophic lateral sclerosis (ALS)	Muscle weakness, muscular atrophy, spasticity, and eventually paralysis.	Motoneurons of the spinal cord, cortex and brain stem.	Intraneuronal aggregates including Bunina bodies, ubiquitinated inclusions, neurofilament-rich "hyaline conglomerate inclusions".	SOD1	[108-111]

PINK1, PTEN-induced putative kinase 1; LRRK2, leucine-rich repeat kinase 2; SOD1, Cu/Zn-superoxide dismutase 1.

### Weakening of cell calcium buffering capacity

Different populations of neurons are selectively affected in different neurodegenerative diseases. The selective vulnerability of some neuronal populations can be explained in part by the decreased expression of some calcium buffering proteins including calbindin-D28K, calmodulin, and parvalbumin. Calbindin-D28K and parvalbumin are absent in motoneuron populations lost early in ALS (e.g. cortical and spinal motoneurons, lower cranial nerve motoneurons), while motoneurons rarely affected or damaged late during the disease express markedly higher levels of these calcium-buffering proteins [110]. The importance of calcium buffering protein levels for calcium homeostasis in ALS was confirmed *in vivo *in parvalbumin transgenic mice interbred with mutant copper/zinc superoxide dismutase SOD1 (mSOD1) transgenic mice, an animal model for familial ALS. These mice present a significant reduction in motoneuron loss, had a delayed disease onset, and a prolonged survival when compared with mSOD1 transgenic mice [113]. Interestingly, a dramatic reduction in calbindin-D28K mRNA and protein levels has been described in the substantia nigra, hippocampus, and nucleus raphe dorsalis in brains of patients with PD [114]. Furthermore, an overall decrease in the levels of calbindin-D28K and calmodulin has been found in the temporal, parietal, and frontal cortex of brains of AD patients [115,116]. In brains of HD patients, a loss of neurons containing calbindin-D28K has also been observed [117]. These findings indicate that the decline in the calcium buffering capacity in brain areas known to be particularly affected in each of these diseases may contribute to the neurodegenerative process.

### Increased vulnerability to excitotoxicity

Glutamate is the most abundant excitatory neurotransmitter of the mammalian nervous system. At the neuronal membrane, glutamate binds to ionotropic and metabotropic receptors. Ionotropic receptors include NMDA, kainate, and AMPA receptors. Activation of the ionotropic receptors leads to channel opening at the plasma membrane and permeability to calcium. Functional AMPARs are homo- and hetero-oligomeric assemblies composed of various combinations of 4 possible subunits. The calcium permeability of AMPARs differs markedly according to whether the GluR2 subunit is present or not in the protein complex: The presence of GluR2 interfering with the channel permeability to calcium. In contrast with ionotropic receptors, metabotropic receptors are not ion channels but are also regulators of calcium homeostasis by mobilizing calcium from internal stores via GTP binding protein-dependent mechanisms.

Excessive activation of glutamate receptors can result in neuronal dysfunction and cell death, a process called excitotoxicity [118]. Uncontrolled activation of ionotropic receptors leads to an excessive influx of calcium through the plasma membrane, which along with impairments of synaptic activation and neuronal plasticity, activate a number of calcium-dependent enzymes involved in the catabolism of proteins, phospholipids, and nucleic acids, as well as in the synthesis of nitric oxide (NO). These alterations can potentially lead to cell death through different pathways, such as membrane break-down, cytoskeletal alterations, and NO-derived free radical production. Increased activation of glutamate receptors has been described in AD, PD, ALS, and HD.

In the case of ALS, *in vitro *and *in vivo *findings suggest that motoneurons are more vulnerable to AMPAR-mediated excitotoxicity than are other neuronal subclasses [119]. This vulnerability in part reflects the absence of detectable GluR2 subunit in spinal motoneurons implicating that they express calcium-permeable AMPARs [120,121]. Excitotoxicity appears to be also involved in familial cases of ALS where mutations in the SOD1 gene are found. Motoneurons from the SOD1 mutant mice exhibit greatly increased vulnerability to glutamate toxicity by AMPARs, which are associated with sustained elevations of intracellular calcium levels, enhanced oxyradical production, and mitochondrial dysfunction [122].

Many lines of evidence support a role of excessive activation of glutamate receptors by excitatory amino acids in the pathogenesis of HD. Neurons expressing high levels of NMDAR are lost early in the striatum in HD individuals, and injection of NMDAR agonists into the striatum of rodents or non-human primates recapitulates the pattern of neuronal damage observed in HD [106]. Interestingly, Sun and colleagues showed that polyQ expansions (^exp^) in huntingtin (Htt) may promote glutamate-mediated excitotoxicity. Normal Htt inhibits channel receptor activity by binding to NMDA and kainate receptors via post synaptic density 95 (PSD-95). The binding of PSD-95 to NMDA or kainate receptors causes the clustering of the receptors at the postsynaptic membrane to regulate NMDA-dependent LTP and LTD. Htt^exp ^was found to interfere with the ability of Htt to interact with PSD-95, causing sensitization of NMDAR and thus promoting neuronal apoptosis induced by glutamate [123].

PD is characterized by a loss of dopamine-containing neurons in the substantia nigra pars compacta. During nigrostriatal dopaminergic depletion, the glutamatergic projections (from subthalamic nucleus to the basal ganglia output nuclei) become overactive [124]. Several studies have shown that a pharmacological inhibition of glutamatergic neurotransmission can ameliorate motor abnormalities in experimental models of PD [125-128]. Alterations of NMDAR functions in PD include impairments in binding of several NMDAR ligands, including L-glutamate or the NMDAR interacting antagonists CPP and MK-801 [102]. Other impairments of NMDAR function in PD include alterations in NMDAR subunits gene expression, protein abundance, and phosphorylation [102].

Excitotoxicity also appears to play an important role in the pathogenesis of AD. The AD brain is characterized by the presence of lesions, including senile plaques that are mainly formed by aggregated amyloid-β (Aβ) peptides [97,129]. Aβ seems to be able to increase the vulnerability of neurons to excitotoxicity mediated by NMDAR [96,130]. Aβ oligomers have been found to cause an increase in NMDAR activity, which might require direct association between Aβ oligomers and the NR1 subunit of NMDAR [131]. There is also evidence that glutamate or other endogenous glutamate receptor agonists are increased in AD. Furthermore, NMDAR subunits NR1, NR2A, and NR2B protein levels and phosphorylation are selectively reduced in AD and these abnormalities correlate with the cognitive impairments [132]. Finally, it is important to note that memantine, a low- to moderate-affinity NMDAR antagonist, is approved for the treatment of moderate to severe AD. Clinical trials have shown that memantine treatment leads to functional improvement in AD patients [133].

### Deregulation of calcium channels

The deregulation of calcium channels other than glutamate receptors is also described in some neurodegenerative diseases. Studies have demonstrated that autoimmunity may be involved in ALS pathogenesis [134]. Delbono and colleagues have shown that most patients with sporadic ALS possess immunoglobulins (IgGs) directed against VGCCs and that the titers of these antibodies correlate with disease progression rates [135,136]. Interestingly, ALS IgGs passively transferred into mice selectively increased intracellular calcium and produced similar ultrastructural changes in motoneurons than those observed in ALS [137,138]. These results support the idea that calcium is central in the selective vulnerability of motoneurons in ALS. It is important to note, however, that the notion of autoimmunity in ALS remains controversial [139,140].

Tang and collaborators have demonstrated that Htt^exp ^can facilitate InsP3R activity. The authors showed that there are functional interactions at the molecular and cellular levels between Htt and InsP3R. The expended version of Htt binds to InsP3R much stronger than the wild type Htt. This biochemical association is correlated with increased sensitivity of InsP3R to InsP3 in single channel recordings of isolated receptors exposed to Htt^exp ^and in measurements of calcium transients in medium spiny neurons transfected with Htt^exp^. The authors have suggested that Htt directly interacts with the InsP3R1 cytosolic carboxy-terminal tail and that binding to this limited region of InsP3R1 is highly dependent on the presence of polyQ expansions within Htt [141].

Alterations in calcium homeostasis in some neurodegenerative diseases could also be the consequence of mutations in ion channels. Our group has recently identified and characterized a new calcium channel involved in AD pathogenesis [142]. We have showed that a polymorphism in the gene coding for CALHM1 leads to a decrease in the activity of the channel and is genetically associated with the risk of developing late-onset AD in large European populations (see section "*CALHM1: A novel regulator of calcium homeostasis and AD pathogenesis"*). In ALS patients, a genome-wide association study identified the inositol 1,4,5-triphosphate receptor 2 gene (ITPR2) as a candidate susceptibility gene [143,144]. ITPR2 is a main regulator of intracellular calcium concentrations. It is also important to mention that spinocerebellar ataxia type 6 (SCA6) is a neurodegenerative disorder caused by CAG repeat expansions within the P/Q type calcium channel CaV2.1 gene and is characterized by predominant degeneration of cerebellar Purkinje cells [145-148].

### Disruption of mitochondrial calcium homeostasis

Mitochondria are important calcium regulators. Indeed, mitochondria can take up calcium by an energy-dependent process through the uniporter and thus reduce cytosolic calcium concentrations. Mitochondria can also release their calcium content through a Na^+^/Ca^2+ ^exchanger and the mitochondrial permeability transition (MPT) pore upon appropriate stimulation. Various factors, including high mitochondrial calcium levels and mitochondrial depolarization, trigger MPT pore opening. Prolonged opening of this pore has been associated with the release of apoptotic factors and cell death. Malfunctions of mitochondria have been associated with various neurodegenerative diseases. Excitotoxity is one mechanism that eventually leads to mitochondrial dysfunction and cell death. Other mitochondrial defects independent of excitotoxicity have also been found [149].

A clear connection has been established between mitochondria, calcium, and neurodegeneration in HD. Panov and colleagues have found that lymphoblast mitochondria from patients with HD have a lower membrane potential and depolarize at lower calcium loads than mitochondria from controls do. A similar defect in brain mitochondria from transgenic mice expressing mutant Htt has been described [150,151]. The authors suggest that mitochondrial abnormalities may be due to a direct binding of mutant Htt on the organelle in neurons [150]. Mutant Htt interaction with mitochondria has later been confirmed and a recombinant truncated mutant Htt protein was found to directly induce MPT pore opening in isolated mouse liver mitochondria. Importantly, the mutant Htt protein significantly decreased the calcium threshold necessary to trigger MPT pore opening [152]. A marked decrease in mitochondrial complex II activity (succinate dehydrogenase, SD) has been found in the brains of HD patients. This inhibition of mitochondrial complex II induces a long-term potentiation of NMDA-mediated synaptic excitation in the striatum and is dependent on dopamine [153].

In sporadic ALS, defective electron transport chain function, perturbed mitochondrial calcium buffering, oxidative stress, and altered mitochondrial ultra-structure have been observed [154]. Mitochondrial abnormalities have also been found in patients with ALS and in transgenic mice with mutant SOD1 [155,156]. In G93A SOD1 mutant transgenic mice, a significant decrease in mitochondrial calcium loading capacity in brain and spinal cord was found [157]. Mutant SOD1 may also affect intracellular calcium levels through a direct toxic effect on mitochondria [158,159].

Defects in mitochondria have also been observed in AD. Mitochondria from AD patient fibroblasts take up less calcium compared to normal controls and they sequester more calcium following oxidative stress [160]. Pyramidal hippocampal neurons show increased levels of mitochondrial DNA and proteins in the cytoplasm and lysosomes [161]. Besides, the Aβ peptide was found in mitochondria from brains of transgenic mice and AD patients and is associated with diminished enzyme activity of the respiratory chain complexes III and IV, and with a reduction in the rate of oxygen consumption [162]. Recent evidence also indicates that Aβ can be transported into mitochondria via the translocase of the outer membrane TOM [163]. Furthermore, Aβ oligomers were proposed to induce a mitochondrial calcium overload resulting in a massive calcium influx and toxicity in neurons [164]. Mitochondrial Aβ accumulation in AD neurons may thus potentiate calcium-induced opening of the MPT pore and mitochondrial swelling [165,166].

Strong evidence also implicates mitochondria impairments in PD pathogenesis. Dysfunctional mitochondria with reduced activities of complex I and of NADH cytochrome c reductase in neurons from the substantia nigra have been observed in PD [167]. Evidence that mitochondria defects are involved in PD also comes from toxic substances known to induce Parkinson-like symptoms, such as MPTP and rotenone [168-170]. The active metabolite of MPTP, the 1-methyl-4-phenylpyridinium ion (MPP+), is an inhibitor of the mitochondrial electron transport chain complex I and a substrate of the dopamine transporter. Thus, MPP+ can accumulate in dopaminergic neurons, where it confers toxicity and leads to neuronal death through complex I inhibition. This has many deleterious consequences, including increased free radical production, oxidative stress, and decrease ATP production, causing increased intracellular calcium concentrations, excitotoxicity, and NO-related cellular damage. Likewise, rats administered with the highly selective complex I inhibitor rotenone developed a PD-like syndrome characterized by neuronal degeneration and formation of α-synuclein rich inclusion bodies [171].

## Calcium homeostasis deregulations in AD

A large body of literature supports the notion that a deranged intracellular calcium signaling occurs in AD and may be involved in both APP processing deregulation and neurodegeneration [7,99,172]. Several *in vitro *and *in vivo *studies have identified calcium signaling pathways relevant to AD pathogenesis.

Recently, two studies have addressed the important question of neuronal calcium dynamics *in vivo *in the brain of APP-expressing mouse models. Using brain injections of calcium indicator dyes, the authors observed a calcium overload in dendritic spines [173] and an increase in the frequency of spontaneous calcium transients [174] in neurons adjacent to cortical amyloid plaques. Kuchibhotla and colleagues showed that senile plaques impaired neuritic calcium homeostasis *in vivo *and result in a structural and functional disruption of neuronal networks [173]. Busche and colleagues used double transgenic APP23xPS45 mice to visualize *in vivo *amyloid plaques and cortical neurons. They sequentially injected the calcium indicator dye Oregon Green 488 BAPTA-1 AM in order to monitor calcium transient and firing of action potentials in neurons and thioflavin S to label fibrillar amyloid deposits. In the transgenic mice, they found that only half of the neurons were active in the normal frequency range, whereas the remaining neurons were either silent or hyperactive. They further demonstrated that these hyperactive neurons were found in close proximity to the plaque borders, whereas the proportion of silent cells gradually increases at greater distances from the plaques. They observed a strict correlation between the formation of amyloid plaques, the appearance of hyperactive neurons, and the impairment of the animal's learning capability [174]. These data are important because they provide *in vivo *evidence for a calcium-dependent mechanism of disturbed neuronal and synaptic function in AD. They also represent an intriguing observation because calcium homeostasis in neurons and in dendritic spines supports neural networks directly relevant to learning and memory.

Specific soluble oligomeric species of Aβ were found to be synaptotoxic by affecting LTP and memory in rodents [175-179]. Several studies demonstrated that Aβ-induced synaptic dysfunction is linked to altered calcium signaling. Exposure of cortical neurons to Aβ peptides destabilized calcium homeostasis and rendered neurons more vulnerable to excitoxicity [130,180]. Application of Aβ40 to cortical synaptosomes and cultured neurons was also found to increase calcium influx via activation of VGCCs [181]. CaMKII autophosphorylation and subsequent phosphorylation of the AMPAR GluR1 subunit was strongly inhibited by application of Aβ42 peptides during hippocampal LTP [182]. Furthermore, soluble oligomeric Aβ isolated from AD patient brains was proposed to be toxic by affecting mGluRs and NMDARs [183]. Similar to α-synuclein, Aβ adopts a β-sheet structure and was also proposed to act by forming calcium pores [100,184-187]. Together, these studies support the notion that defects in calcium-dependent synaptic activity are likely to be responsible for the neurotoxic effect of Aβ. Although proximity to senile plaques, which mostly contain deposited Aβ, seems to affect calcium-dependent synaptic functions *in vivo*, it is conceivable to believe that plaques might constitute a reservoir for soluble Aβ species, including oligomeric neurotoxic species. The "calcium poisoning" effect observed *in vivo *in neurons surrounding the amyloid deposits might therefore be due to released soluble neurotoxic oligomeric Aβ species.

### APP, tau, and calcium homeostasis

A sustained increase in intracellular calcium can, *per se*, lead to neurodegeneration and cell death. However, calcium homeostasis deregulation can also affect the fate of proteins involved in the pathogenesis of the disease, as it is the case for tau and Aβ in AD. In addition to the Aβ plaques, histopathological studies of the AD brain have revealed the presence of dramatic ultrastructural changes triggered by other lesions, the neurofibrillary tangles, which consist of aggregated hyperphosphorylated tau proteins [97,129,188]. The state of tau phosphorylation and proteolysis can be regulated by calcium-dependent mechanisms. CaMKII can phosphorylate tau [189]. Cyclin-dependent kinase 5 (cdk5), another kinase involved in tau phosphorylation [190], is indirectly activated by the calcium-activated protease calpain. Indeed, cdk5 has to be associated with its regulatory subunit, p35 to be activated. Conversion of p35 to p25 deregulates cdk5 activity, resulting in an increased cdk5 kinase activity [191]. Calpain cleaves p35 into p25, and thus controls cdk5 activation [192]. Furthermore, tau is dephosphorylated by the calcium/calmodulin-dependent phosphatase, calcineurin [193]. Calpain was also proposed to directly participate in tau proteolysis and degradation [194]. Interestingly, inhibition of calpains was recently found to improve memory and synaptic transmission in a mouse model of AD [195]. Pharmacological inhibition of calpains restored normal synaptic function in both hippocampal cell cultures and hippocampal slices from APP/PS1 mice. Calpain inhibition also improved spatial working memory and associative fear memory in these mice. These beneficial effects of calpain inhibition were associated with restoration of normal phosphorylation levels of CREB [195]. Interestingly, overexpression of tau-tubulin kinase-1 (TTBK1), a kinase phosphorylating tau and activating calpain I and cdk5, led to the formation of neurofilament aggregates and age-dependent memory impairments in a transgenic animal model [196]. The authors also determined that TTBK1 expression resulted in a downregulation of hippocampal NMDAR NR2B subunits, suggesting a potential cross-talk between tau phosphorylation, calcium homeostasis deregulations, and memory deterioration.

Sequential endoproteolysis of APP (amyloid-β precursor protein) by the aspartyl protease β-secretase/BACE1 and by the presenilin/γ-secretase complex leads to the production of Aβ [197,198]. In an alternative non-amyloidogenic pathway, APP is endoproteolyzed within the Aβ region by α-secretase to generate the soluble N-terminal fragment sAPPα, a protein that might possess some neuroprotective properties [198]. APP processing can be regulated by calcium signaling and inversely, APP metabolism can influence calcium signaling. It was shown that calcium influx mediated by calcium ionophores or by membrane depolarization and channel opening, leads to increased production of Aβ [199,200]. Conversly, inhibition of SERCA pumps with thapsigargin and increased cytosolic calcium levels from ER calcium depletion, diminished Aβ generation [201]. It seems that the effect of calcium on APP processing might depend on the source of the ions and on the mechanism involved. Calcium has also been involved in the stimulation of sAPP release [142,201,202]. Moreover, sAPPα can cause a rapid and prolonged reduction in intracellular calcium concentrations and can protect neurons against glutamate neurotoxicity by raising the excitotoxic threshold [180,203]. In addition, carboxy-terminal fragments (CTs) of APP can disrupt calcium homeostasis and render neuronal cells vulnerable to excitotoxicity. It was found that a recombinant CT105 of APP could inhibit Na^+^/Ca^2+ ^exchanger activity in SK-N-SH cells [204] and rendered SK-N-SH cells and rat primary cortical neurons more vulnerable to glutamate-induced excitotoxicity [205]. Importantly, both Aβ and CT105 have been shown to shorten the duration of high frequency stimulation-induced LTP in rat hippocampus *in vivo *[206]. Moreover, adult transgenic mice expressing the CT104 of APP demonstrated spatial learning deficits and defects in the maintenance of LTP [207]. Calcium signaling alterations are also observed in presenilin-1-/- and APP-/- cells and can be reversed by reintroduction of AICD, the C-terminal domain of APP produced by γ-secretase. This suggests that AICD could also be involved in the regulation of calcium signaling [208].

### Presenilins in calcium homeostasis

Presenilins (PSs) are multipass transmembrane proteins involved in many cases of early-onset familial AD (FAD) and represent the catalytic core of the γ-secretase activity [197]. In 2006, it has been proposed that PS holoproteins have calcium leak properties at the ER membrane. The authors showed that PSs could form low-conductance divalent-cation-permeable ion channels in planar lipid bilayers, a property significantly reduced by several FAD-linked PS mutations [209,210]. Furthermore, it has been shown that PSs can physically interact with the InsP3 [211] and ryanodine [212] receptors and facilitate their calcium gating activity at the ER membrane. Overexpression of several FAD PS mutants consistently generated a robust increase in the gating of InsP3R, as compared to wild type PSs [211], suggesting that the pathogenic mutations exaggerate cellular calcium signaling in response to ligand activation. The ER calcium pump SERCA was also found to form a protein complex with endogenous PSs, an interaction that influences SERCA function by controlling ER calcium sequestration and InsP3-mediated calcium liberation [213]. Several of these studies have also proposed that increased ER calcium release via InsP3Rs or RyRs or via modulation of SERCA may modulate APP processing and facilitate Aβ accumulation. Together these studies indicate that disease-linked PS mutations could impact APP metabolism by affecting ER calcium dynamics at multiple levels (Figure 2). Nevertheless, APP metabolism involves a complex series of events and the direct influence of calcium signaling on this process remains to be clearly addressed [7].

### CALHM1: A novel regulator of calcium homeostasis and AD pathogenesis

**Figure 3:**
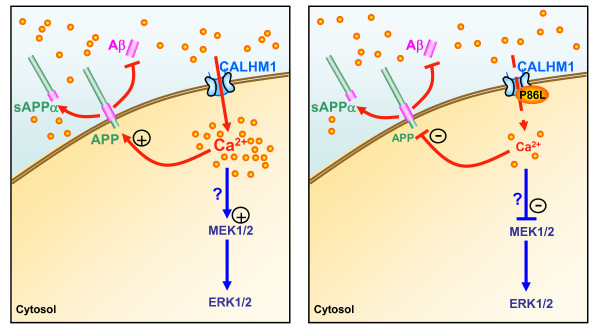
**The role of CALHM1 in calcium signaling: Relevance for APP metabolism and AD pathogenesis**. CALHM1 is a cell surface protein of neuronal origin that shares sequence similarities with NMDAR. CALHM1 expression generates a calcium-selective cation current at the plasma membrane and controls cytosolic calcium concentrations, a mechanism that may lead to ERK1/2 activation (left panel). Importantly, the P86L mutation in CALHM1, which we found associated with an increased risk for AD in European populations, leads to an inhibition of the control of APP processing by CALHM1 (right panel). Consequently, the P86L mutation leads to a derepression of the effect of CALHM1 on Aβ accumulation and thus to an increase of Aβ levels. P86L-CALHM1 promotes Aβ accumulation via a loss of CALHM1 control on calcium permeability and cytosolic calcium levels [142]. Therefore, CALHM1 is a component of a novel cerebral calcium channel family involved in calcium signaling and Aβ metabolism, and thus may represent an important player in the calcium homeostasis deregulations observed in AD pathogenesis.

A number of neurodegenerative disorders are caused by mutations in genes expressed principally in the central nervous system. This is the case for the brain proteins tau and α-synuclein, which are genetically linked to autosomal dominant forms of frontotemporal dementia [214] and PD [215], respectively. Recently, we postulated that susceptibility to AD could come from genes predominantly expressed in affected brain regions, such as the hippocampus. By using a tissue-expression profiling method to screen for genes predominantly expressed in the hippocampus and located in linkage regions for AD, our group identified the *CALHM1 *gene, located on chromosome 10. *CALHM1 *codes for a protein of neuronal origin that shares sequence similarities with NMDAR and that controls both cytosolic calcium concentrations [142] and ERK1/2 activation [216]. Voltage-clamp analyses further revealed that CALHM1 generates a calcium-selective cation current at the plasma membrane [142]. Importantly, we also determined that the frequency of the rare allele of the rs2986017 SNP (single nucleotide polymorphism) in *CALHM1*, which results in the P86L substitution, is significantly increased in AD cases in five independent cohorts. Further investigation demonstrated the functional significance of the rs2986017 SNP by showing that the P86L polymorphism promotes Aβ accumulation via a loss of CALHM1 control on calcium permeability and cytosolic calcium levels [142]. Together, this work provides strong evidence that CALHM1 is a component of a novel cerebral calcium channel family involved in Aβ metabolism and that *CALHM1 *polymorphisms may influence AD risk. The identification of CALHM1 as a key modulator of calcium homeostasis and Aβ levels provides strong support for the calcium hypothesis of AD. This work is also an important step towards understanding the potential pathological cross talk between calcium signaling disturbances, pathways of Aβ accumulation, and neurodegeneration (Figure 3).

## Conclusion

The central role of calcium signaling in brain functions underlines its potential relevance for neurodegeneration. This notion is now supported by overwhelming evidence in several neurodegenerative disorders of the CNS, such as AD, PD, or HD, and in the motoneuron disorder ALS. The apparent complexity of the different calcium-dependent mechanisms involved in neuronal death in these diseases, reflects the sophistication of the pathways available to control normal calcium homeostasis. This complexity makes the identification of precise and common neurotoxic molecular events between these disorders challenging. Nevertheless, it is likely that a precise definition of the calcium signaling failure in neurodegeneration will one day emerge and facilitate the identification of novel therapeutic targets.

## Competing interests

The authors declare that they have no competing interests.

## Authors' contributions

PM, UDW, and VV wrote the manuscript.
